# Hypertension and Cardiovascular Remodelling in Rats Exposed to Continuous Light: Protection by ACE-Inhibition and Melatonin

**DOI:** 10.1155/2014/703175

**Published:** 2014-07-06

**Authors:** Fedor Simko, Olga Pechanova, Kristina Repova Bednarova, Kristina Krajcirovicova, Peter Celec, Natalia Kamodyova, Stefan Zorad, Jarmila Kucharska, Anna Gvozdjakova, Michaela Adamcova, Ludovit Paulis

**Affiliations:** ^1^Department of Pathophysiology, School of Medicine, Comenius University, Sasinkova 4, 81372 Bratislava, Slovakia; ^2^3rd Clinic of Medicine, School of Medicine, Comenius University, 83305 Bratislava, Slovakia; ^3^Institute of Experimental Endocrinology, Slovak Academy of Sciences, 83305 Bratislava, Slovakia; ^4^Center of Excellence NOREG, 81372 Bratislava, Slovakia; ^5^Institute of Normal and Pathological Physiology, Slovak Academy of Sciences, 81371 Bratislava, Slovakia; ^6^Institute of Molecular Biomedicine, School of Medicine, Comenius University, 81372 Bratislava, Slovakia; ^7^Department of Physiology, School of Medicine, Charles University, 50038 Hradec Kralove 1, Czech Republic

## Abstract

Exposure of rats to continuous light attenuates melatonin production and results in hypertension development. This study investigated whether hypertension induced by continuous light (24 hours/day) exposure induces heart and aorta remodelling and if these alterations are prevented by melatonin or angiotensin converting enzyme inhibitor captopril. Four groups of 3-month-old male Wistar rats (10 per group) were treated as follows for six weeks: untreated controls, exposed to continuous light, light-exposed, and treated with either captopril (100 mg/kg/day) or melatonin (10 mg/kg/day). Exposure to continuous light led to hypertension, left ventricular (LV) hypertrophy and fibrosis, and enhancement of the oxidative load in the LV and aorta. Increase in systolic blood pressure by continuous light exposure was prevented completely by captopril and partially by melatonin. Both captopril and melatonin reduced the wall thickness and cross-sectional area of the aorta and reduced the level of oxidative stress. However, only captopril reduced LV hypertrophy development and only melatonin reduced LV hydroxyproline concentration in insoluble and total collagen in rats exposed to continuous light. In conclusion, captopril prevented LV hypertrophy development in the continuous light-induced hypertension model, while only melatonin significantly reduced fibrosis. This antifibrotic action of melatonin may be protective in hypertensive heart disease.

## 1. Introduction

Left ventricular (LV) hypertrophy, although representing an adaptation to hemodynamic overload, is associated with increased cardiovascular risk [1, 2]. The search for new approaches for the prevention or regression of LV hypertrophy in different models of pathological myocardial growth continues [3–7].

Melatonin (N-acetyl-5-methoxytryptamine), the most abundant secretory product of the vertebrate pineal gland that controls biological rhythms [8], has numerous beneficial actions in the heart [9–12] and plays an important role in the pathogenesis of hypertension [13–16]. Experimental pinealectomy, a procedure that is easily performed in rats [17], reduced the level of both day-time and nocturnal melatonin [8] and resulted in an enhancement of vascular responsiveness to vasoconstriction stimuli [18], increased blood pressure, and myocardial fibrosis [19]. An alternative approach to attenuate melatonin production, associated with a blood pressure rise, is the exposure of rats to continuous 24 hours/day chronic lighting [20]. This model of melatonin-deficient hypertension seems to be more physiological than pinealectomy because it reduces only nocturnal melatonin secretion and no surgery is involved [20]. Since melatonin exerts cardiovascular protection on several levels including antioxidant and scavenging actions [21–23], endothelial protection, sympatholytic effect [24–28], and antiarrhythmic effects [29], it is comprehensible that a deficiency of melatonin could result in pathological alterations in circulation and target organ damage [2, 8]. Indeed, several studies with patients suffering from acute myocardial infarction with ST segment elevation indicated that deficit of melatonin could be a negative prognostic factor of the severity of infarction, subsequent remodelling, and prognosis [30–33] and that melatonin supplementation may exert beneficial effects as a radical scavenger in a human model of myocardial ischemia-reperfusion damage [34, 35].

This study investigated whether continuous light-induced hypertension results in the pathological growth of the heart and the aorta and whether melatonin can modify these potential alterations. Moreover, the effect of melatonin was compared with the angiotensin converting enzyme (ACE) inhibitor, captopril. Since ACE-inhibitors are antihypertensives with well-established antihypertrophic effect, which is also based on inhibition of angiotensin II production and interference with aldosterone, catecholamines, or endothelin production [37, 38], ACE-inhibition may be protective also in continuous light-induced hypertension.

## 2. Material and Methods

### 2.1. Animals and Treatment

Male adult three-month-old Wistar rats (Dobra Voda, Slovak Republic) were randomly divided into four groups (*n* = 10 in each group): control (Wistar) rats (c), rats exposed to 24 hours/day continuous light (24), and continuous light-exposed rats treated with either captopril (100 mg/kg/day; Egis Pharmaceuticals Ltd, Budapest; 24C) or melatonin (10 mg/kg/day; Sigma Chemical Co., Deisenhofen, Germany; 24M). Captopril and melatonin were dissolved in drinking water and their concentration was adjusted to daily water consumption to ensure correct dosage. The natural water consumption of rats was about 12-13 mL/100 g of body weight. In order to ensure that all the amount of water with dissoluted melatonin was really drank by a particular rat, only 10 mL/100 mg of water-melatonin solution was offered. The solution was prepared as follows: 10 mg of melatonin was dissoluted in 100 mL water, while no additional substance was added to dissolute the substance. Melatonin containing solutions were protected from light exposure by using black bottles. All rats were housed in individual cages at 22–24°C and fed a regular pellet diet* ad libitum* in accordance with the* Guide for the Care and Use of Laboratory Animals *published by the US National Institutes of Health (NIH Publication number 8523, revised 1985).

Systolic blood pressure (SBP) was measured each week by noninvasive tail-cuff plethysmography (Hugo-Sachs Elektronic, Freiburg, Germany). After six weeks, rats were decapitated and body weight (BW), heart weight (HW), and LV and right ventricle weights (LVW, RVW) were determined and their relative weights (LVW/BW and RVW/BW ratio) were calculated. Left ventricle samples were frozen at −80°C and later used for the determination of hydroxyproline concentrations and oxidative stress parameters.

### 2.2. Morphometry of Aorta

Thoracic aorta samples were fixed for 24 hours in 4% formaldehyde, embedded in paraffin, cut in serial 5 *μ*m thick sections, and stained with haematoxylin and eosin. Wall thickness (WT) in *μ*m and the inner circumference in mm were measured using light microscopy and a two-dimensional image analyzer (Impor Pro; Kvant s.r.o., Bratislava, Slovak Republic). Inner diameter (ID) expressed in mm and cross-sectional area (CSA) expressed in mm^2^ were then calculated [28]. During morphometric measurements the observer was blinded (only the numbers of animals were accessible for the observer).

### 2.3. Determination of Hydroxyproline

Collagenous proteins were isolated according to Pelouch et al. [39, 40]. Hydroxyproline concentrations were analyzed spectrophotometrically at 550 nm [41].

### 2.4. Oxidative Load Measurement

Tissue samples were homogenized in 1 mL phosphate buffered saline and centrifuged at 19320 g (4°C) for 5 minutes and the supernatant was used for further biochemical analyses.

Advanced glycation end-products (AGEs) as markers of carbonyl stress were measured using the characteristic fluorescence (*λ*ex. = 370 nm, *λ*em. = 440 nm) [42]. Briefly, tissue homogenates were diluted 10-fold with phosphate buffered saline and the AGEs concentration was calculated based on the calibration curve prepared with AGE-BSA calibrator [43].

Advanced oxidation protein products (AOPP) were determined spectrophotometrically [44]. Briefly, 200 *μ*L of appropriately diluted tissue homogenates were incubated with glacial acetic acid for 2 minutes and the absorbance was read at 340 nm using Sapphire II instrument (Tecan, Grödig, Austria). AOPP concentration was calculated on the basis of the calibration curve of chloramin T with potassium iodide. Proteins were quantified using BCA protein assay kit (Sigma Aldrich, Steinheim, Germany) [42].

### 2.5. CoQ and Tocopherol Measurement

Concentrations of CoQ9ox, CoQ10ox, and gamma- and alpha-tocopherol were determined by HPLC method according to Lang et al. [45] with some modifications [46]. Concentrations of CoQ and tocopherols were detected spectrophotometrically at 275 nm and 295 nm, respectively.

### 2.6. Statistical Analysis

Results are expressed as mean ± S.E.M. One-way, two-tailed analysis of variance (ANOVA) and the Bonferroni test were used for statistical analysis. Differences were considered significant if the *P* value <0.05.

## 3. Results

### 3.1. Cardiovascular Parameters

**Figure 1:**
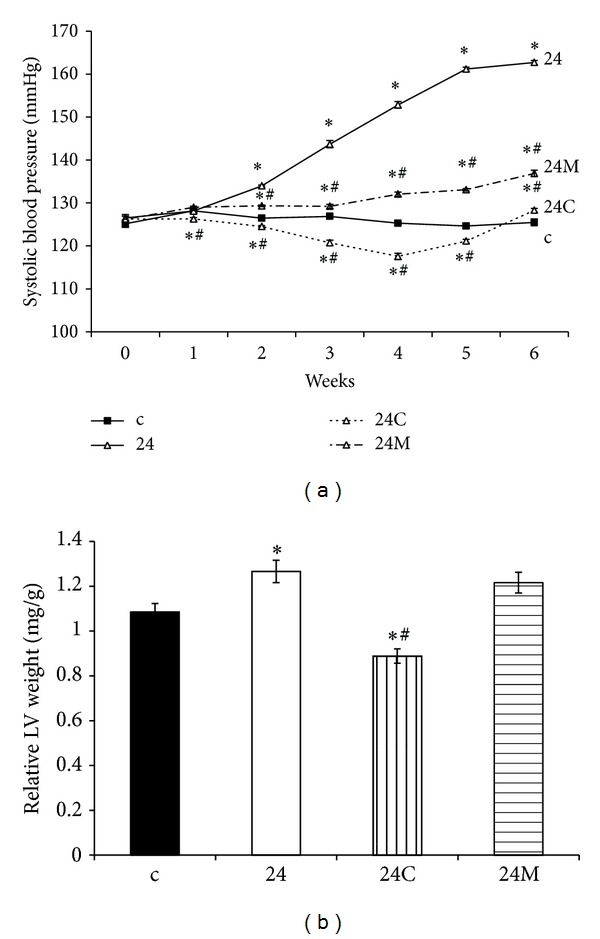
The influence of captopril (24C) and melatonin (24M) on blood pressure (a) and relative left ventricle (LV) weight (LVW/BW) (b) in 24 hours/day continuous light exposure-induced hypertension (24). c: Wistar controls. *P < 0.05 versus c; ^#^ *P* < 0.05 versus 24.

SBP was 125 ± 0.79 mmHg in control rats and 163 ± 0.48 mmHg in continuous light-group. SBP decreased significantly (*P* < 0.05) by both captopril (21%) and melatonin (15%) treatment compared to continuous light-group (Figure 1(a)). The LVW/BW ratio after six weeks of treatment was 1.08 ± 0.04 mg/g and 1.27 ± 0.05 mg/g, in the control and continuous light-group, respectively. Captopril decreased the LVW/BW ratio (30%, *P* < 0.05), while melatonin had no effect (Figure 1(b)). Absolute left ventricular weight was also affected only by the captopril treatment (Table 1).

### 3.2. Morphometry of Aorta

**Figure 2:**
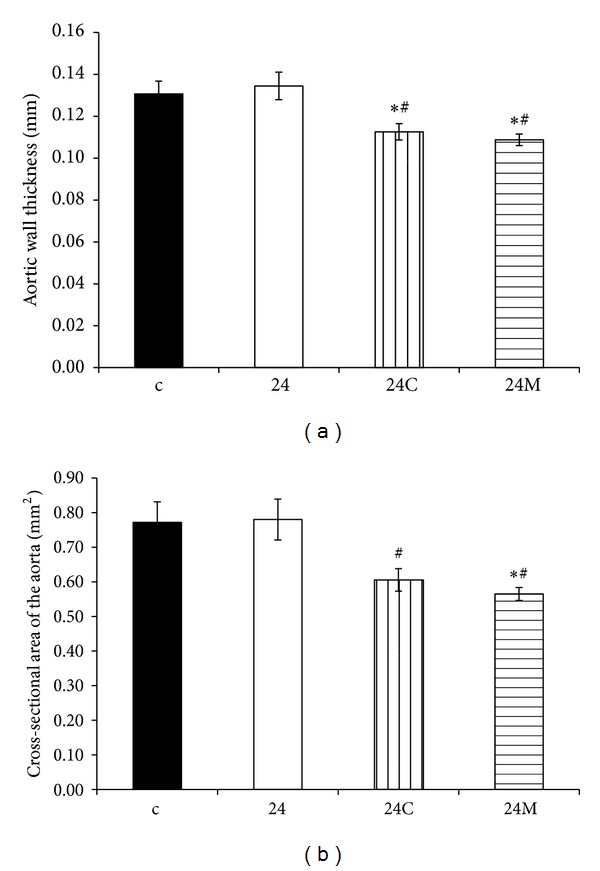
The influence of captopril (24C) and melatonin (24M) on wall thickness (a) and cross-sectional area (b) of the aorta in 24 hours/day continuous light exposure-induced hypertension (24). c: Wistar controls. *P < 0.05 versus c; ^#^ *P* < 0.05 versus 24.

The WT of aorta was 0.131 ± 0.006 mm in controls and 0.135 ± 0.006 mm in continuous light-group. Both captopril and melatonin reduced the WT of aorta (18%, *P* < 0.05 and 19%, *P* < 0.05, resp.) (Figure 2(a)). The cross-sectional area of aorta was 0.772 ± 0.059 mm^2^ in controls and 0.780 ± 0.059 mm^2^ in the continuous light-group. Both captopril and melatonin reduced the cross-sectional area of the aorta (22%, *P* < 0.05 and 28%, *P* < 0.05, resp.) (Figure 2(b)).

### 3.3. Hydroxyproline in the Soluble and Insoluble Collagen and Total Hydroxyproline in the LV

**Figure 3:**
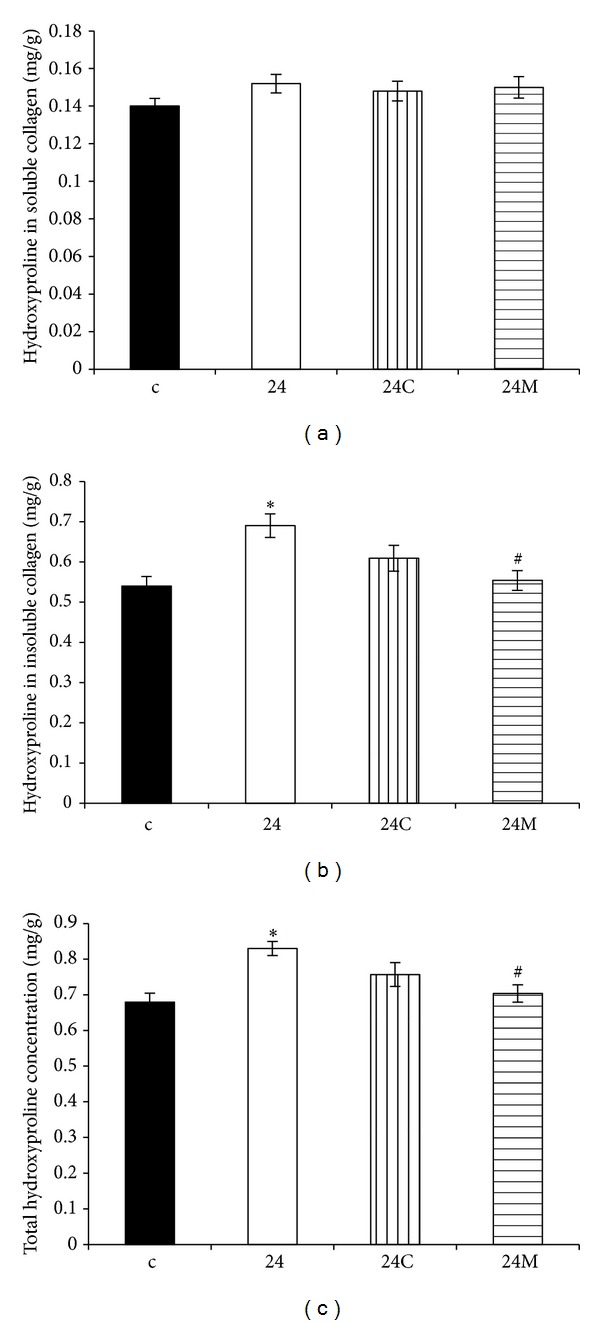
The influence of captopril (24C) and melatonin (24M) on hydroxyproline concentration in soluble (a) and insoluble (b) collagen proteins and on total hydroxyproline concentration (c) in 24 hours/day continuous light exposure-induced hypertension (24). c: Wistar controls. *P < 0.05 versus c; ^#^ *P* < 0.05 versus 24.

Hydroxyproline concentration in the soluble collagenous protein was 0.14 ± 0.004 mg/g and 0.152 ± 0.005 mg/g in the control and continuous light-groups, respectively. Neither captopril nor melatonin had any effect on hydroxyproline concentration in soluble collagen (Figure 3(a)).

Hydroxyproline concentration in the insoluble collagen was 0.540 ± 0.024 mg/g and 0.690 ± 0.029 mg/g in the control and continuous light-groups, respectively, and it was significantly reduced only by melatonin (20%, *P* < 0.05) (Figure 3(b)).

Total hydroxyproline concentration was 0.68 ± 0.025 mg/g and 0.830 ± 0.020 mg/g in the control and continuous light-groups, respectively, and it was reduced only by melatonin (15%, *P* < 0.05) (Figure 3(c)).

### 3.4. Oxidative Stress Parameters

**Figure 4:**
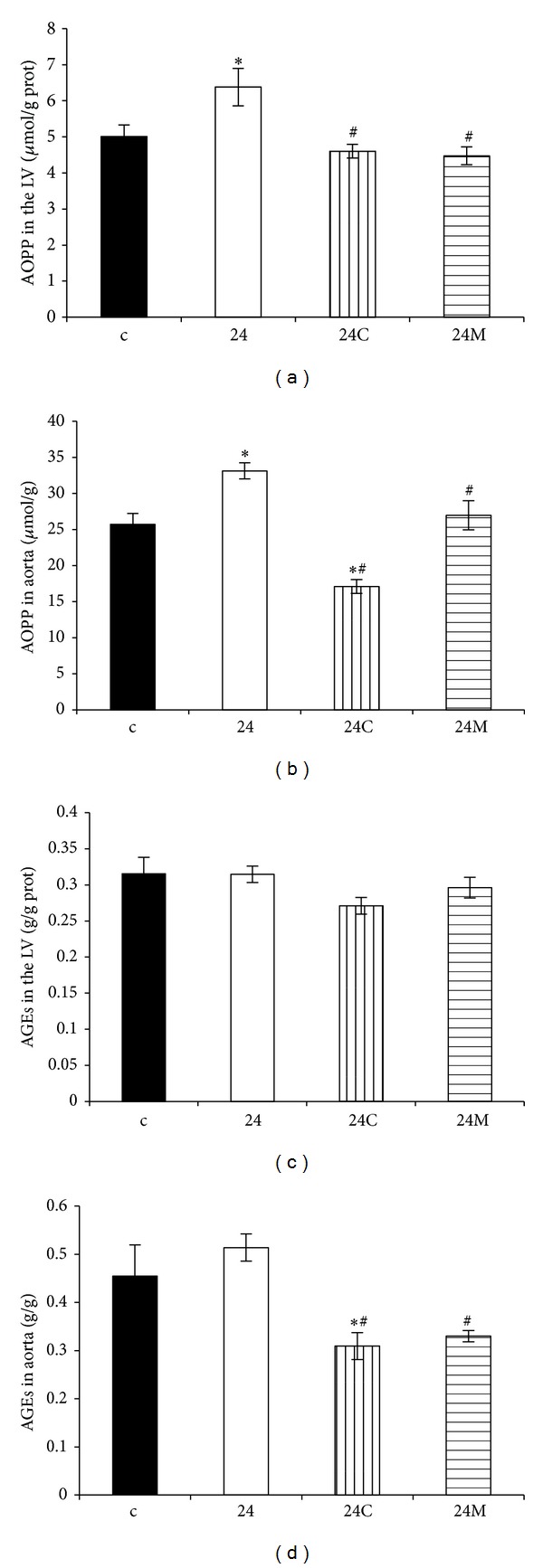
The influence of captopril (24C) and melatonin (24M) on advanced oxidation protein products (AOPP) in the LV (a) and aorta (b) and on advanced glycation end-products (AGEs) in the LV (c) and in the aorta (d) in 24 hours/day continuous light exposure-induced hypertension (24). c: Wistar controls. *P < 0.05 versus c; ^#^ *P* < 0.05 versus 24.

The LV AOPP was 5.01 ± 0.32 *μ*mol/g prot. and 6.38 ± 0.52 *μ*mol/g prot. in the control and continuous light-groups, respectively, and both captopril and melatonin significantly reduced AOPP in the LV (28%, *P* < 0.05 and 30%, *P* < 0.05, resp.) (Figure 4(a)). The aortic AOPP was 25.72 ± 1.52 *μ*mol/g prot. and 33.11 ± 1.10 *μ*mol/g prot. in the control and continuous light-groups, respectively, and both captopril and melatonin significantly reduced AOPP in the aorta (48%, *P* < 0.05 and 19%, *P* < 0.05, resp.) (Figure 4(b)).

The aortic AGEs were 0.43 ± 0.06 and 0.47 ± 0.04 in the control and continuous light-groups, respectively. Both captopril and melatonin significantly reduced AGEs in the aorta (34%, *P* < 0.05 and 30%, *P* < 0.05, resp.) (Figure 4(d)).

### 3.5. CoQ and Tocopherol

The left ventricular CoQ9ox, CoQ10ox, and alpha-tocopherol levels were not changed in either group. Gamma-tocopherol concentration in the LV was decreased in the continuous light-group and neither captopril nor melatonin modified it (Table 2).

## 4. Discussion

Under physiological conditions in humans, melatonin concentration is low during the day, but it is much higher at night and is accompanied by lowering of the nocturnal blood pressure [47]. The evening rise in melatonin represents an important part of suprachiasmatic adaptation to the rest and activity periods [48, 49]. Inhibition of the nighttime rise in melatonin by continuous light exposure in rats [20] mimics the melatonin profile of patients with nondipping blood pressure profile [26], in which an insufficient rise in 6-sulphamethoxymelatonin in urine reflects a blunted increase in serum melatonin during the night period [50]. This functional pinealectomy in rats results in gradually developing hypertension [51], although the blood pressure rise is less pronounced than in L-NAME [37, 52] or spontaneously hypertensive rat (SHR) [38] models of experimental hypertension.

**Figure 5:**
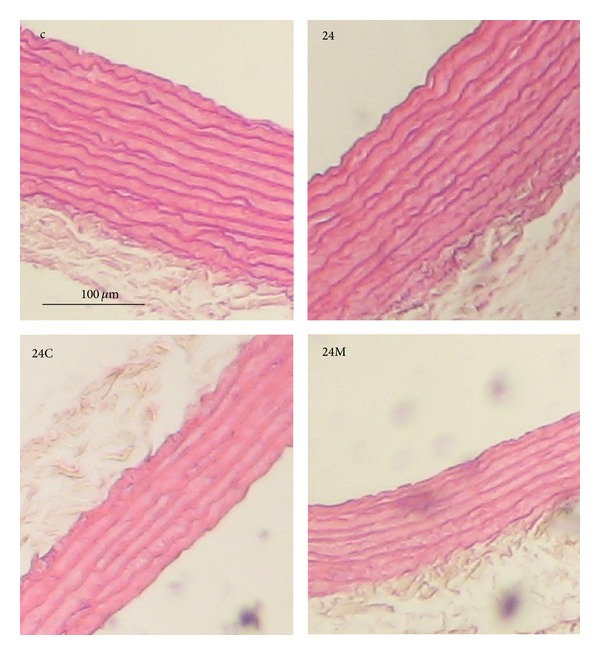
The influence of captopril (24C) and melatonin (24M) on wall thickness of aorta stained by hematoxylin-eosin in 24 hours/day continuous light exposure-induced hypertension (24). c: Wistar controls.

In rats exposed to constant lighting, hypertension development was associated with a rise in the LV weight, whereas no changes in the wall thickness and cross-sectional area of the aorta were observed. Interestingly, captopril inhibited the rise in blood pressure completely, reduced the LV weight even bellow the control values, similarly as was shown in SHR model [38], and reduced the aortic wall thickness and cross-sectional area (Figures 2(a), 2(b), and 5). Melatonin, like captopril, also reduced blood pressure and the aortic wall thickness and cross-sectional area (Figures 2(a), 2(b), and 5). However, unlike captopril, melatonin had no effect on the weight of the LV, similarly as has been reported in L-NAME-hypertension [53]. These observations suggest that hypertrophic growth of the LV and of the aorta are differently controlled in this particular model.

**Figure 6:**
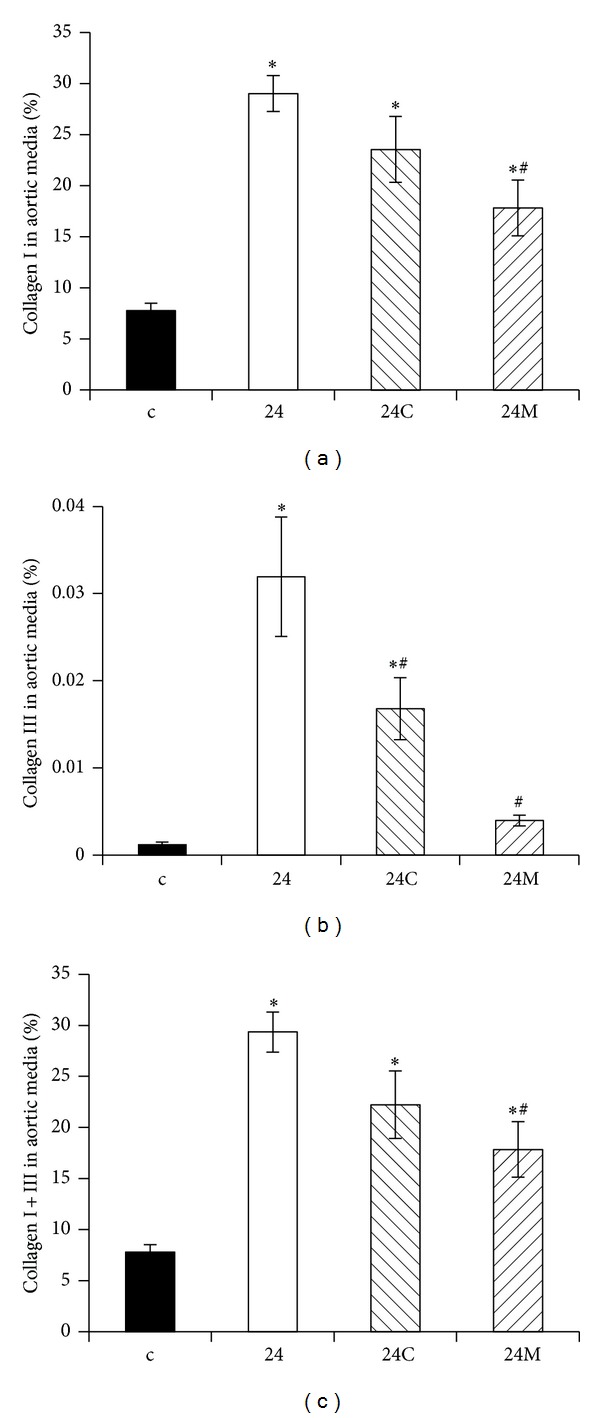
The influence of captopril (24C) and melatonin (24M) on the concentration of collagen I (a), III (b), and the sum of collagens I and III (c) in the aortic media in 24 hours/day continuous light exposure-induced hypertension (24). c: Wistar controls. *P < 0.05 versus c; ^#^ *P* < 0.05 versus 24 (according to [36] with permission).

**Figure 7:**
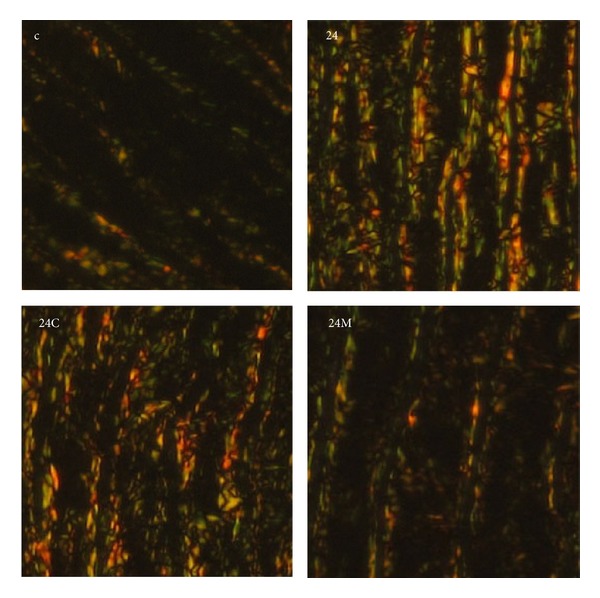
The influence of captopril (24C) and melatonin (24M) on the concentration of collagen in the aortic media in 24 hours/day continuous light exposure-induced hypertension (24). Specimens are stained with picrosirius red and evaluated in polarized light: red to yellow color represents collagen I and green color represents collagen III. c: Wistar controls (according to [36] with permission).

Fibrotic LV remodelling after six-week exposure to constant light was characterized by elevated hydroxyproline levels in the insoluble (maturated, more cross-linked) and in total collagen (the sum of soluble and insoluble collagens). Only melatonin significantly reduced hydroxyproline concentration in both insoluble and total collagen. In a previous experiment with a combination of continuous light plus L-NAME model of hypertension analogical protection by melatonin was achieved [51]. Despite the increase of blood pressure and that fibrosis was even more pronounced in a continuous light plus L-NAME hypertension than in the continuous light model, melatonin reduced blood pressure and fibrosis in a similar way as in this experiment and also without attenuation of LV hypertrophy [51]. Moreover, this fibrosis-reducing effect of melatonin was confirmed in SHR model [38]. The indolamine also reduced the accumulation of collagens I and III in the aorta in continuous light-induced hypertension [36] (Figures 6 and 7). Since the quality rather than the quantity of the hypertrophied myocardium is important, while fibrosis is one of the decisive negative prognostic factors [54], this antifibrotic effect of melatonin may be of importance in patients with hypertensive heart.

Different impacts of melatonin and captopril treatment on the LV hypertrophy and LV fibrosis need to be addressed. It is generally accepted that LV hypertrophy development or its regression is mainly determined by the level of haemodynamic burden. Thus, the fact that only captopril reduced LV weight may be driven by a more prominent reduction in blood pressure by captopril than by melatonin. The distinct effects of captopril and melatonin on the LV fibrosis may be related to the complexity of hypertension development in this particular model and by the pleiotropic actions of both drugs. Fibrosis development is mainly dependent on the humoral disbalance between oxidative load, pro-proteosynthetic, and pro-proliferative factors such as angiotensin II, aldosterone, endothelin, or catecholamines on the one side and substances with growth attenuating potential, such as nitric oxide (NO), prostacyclin, or atrial natriuretic peptide [54–56] on the other side. Continuous light-induced hypertension development is obviously associated with more complex neurohumoral disequilibrium than with a simple deficit of the melatonin. The plasmatic renin activity was increased five weeks after pinealectomy, possibly reflecting an increased sympathetic tone, a major contributor to hypertension development in pinealectomized animals [57]. Constant illumination was associated with enhanced adrenocorticotropic hormone and corticosterone plasmatic concentrations [58]. The pluripotent effects of melatonin and captopril on this neurohumoral activation in light-exposed rats may be responsible for their distinct protective impact. Reduction in blood pressure by melatonin may be due to increased GABA(A)—ergic activity leading to lower plasmatic angiotensin II concentration [59] with sympatholytic [28], oxidative load reducing [60, 61], and NO-promoting [25] effects. However, captopril is able to reduce the level of angiotensin II, aldosterone, and endothelin and the sympathetic drive [62].

The important question is the dose of captopril and melatonin that should be chosen to counteract pathological alterations of the continuous light-induced hypertension and LVH. 100 mg/kg/day of captopril [36, 38, 51] and 10 mg/kg/day of melatonin [38, 51, 53] used in our previous works were shown to lower increased blood pressure and protect the cardiovascular system against deleterious effect of hemodynamic overload. Moreover, the higher dose of captopril (150 mg/kg/day), which was tested in our pilot studies, was not well tolerated, as convulsions of striated muscles occurred as a result of hyperkalemia. Analogically, higher doses of melatonin were shown to have sedative effects. Thus, the tolerable pharmacological dose of captopril (100 mg/kg/day) and melatonin (10 mg/kg/day) may be sufficient to induce the potential protective effects of tested substances.

The choice of captopril from the large group of accessible ACE-inhibitors was determined by three points. First, captopril is considered to be the gold standard, since it was the first ACE-inhibitor approved by Food and Drug Administration in 1993 as an effective treatment of heart remodeling and failure. Second, captopril contains SH group with potentially beneficial effect against oxidative stress. Third, in our previous experiments captopril was used [4, 36–38, 51] and thus the presented data of this experiment may be compared with our previous results from other models of hypertension.

It should be emphasized that in this experiment three-month-old rats (which may be considered to be adult) were used in a “preventive” experiment. However, when substantially younger rats with developing LVH and fibrosis were used, the results could be different.

In conclusion, captopril prevented LV hypertrophy development in the continuous light-induced hypertension model but only melatonin significantly reduced fibrosis. This antifibrotic action of melatonin may be protective in hypertensive heart disease.

## 5. Limitations of the Work

In the model of continuous light exposure it would be of benefit to assess blood pressure more accurately by using telemetry system or by making measurements every 12 hours by tail-cuff plethysmography to disclose circadian rhythms of blood pressure. Moreover, it would be useful to investigate the group with continuous light and combination of captopril + melatonin treatment to show the potential complementary protection of both drugs. Furthermore, it may be useful to compare biochemical determination of the collagen level in the LV with the histochemical method, similarly as to know the level of NO_*x*_ (nitrite-nitrate). However, these approaches were beyond the scope and possibilities of this experiment.

## Figures and Tables

**Table 1:** The body weight (BW), right ventricle weight (RVW), relative RVW (RVW/BW), and left ventricle weight (LVW) in control rats (c), rats exposed to 24 h/day continuous light without treatment (24), and rats treated with captopril (24C) and melatonin (24M).

	BW (g)	RVW (mg)	RVW/BW (mg/g)	LVW (mg)
c	442.5 ± 14.9	201.7 ± 10.0	0.45 ± 0.01	478.9 ± 21.1
24	392.8 ± 8.6*	214.7 ± 6.3	0.55 ± 0.02*	498.2 ± 24.8
24C	402.3 ± 11.6	155.1 ± 8.5^∗#^	0.39 ± 0.02^#^	361.9 ± 19.0^∗#^
24M	388.2 ± 11.6*	160.5 ± 10.3^∗#^	0.41 ± 0.03^#^	485.6 ± 13.4

Values are mean ± SEM, *P < 0.05 versus c, ^#^ *P* < 0.05 versus 24.

**Table 2:** The alpha- and gamma-tocopherol, oxidized coenzyme Q9 (CoQ9ox), and oxidized coenzyme Q10 (CoQ10ox) in control rats (c), rats exposed to 24 h/day light without treatment (24), and rats treated with captopril (24C) or melatonin (24M).

	Alpha-tocopherol in the LV (nmol/g)	Gamma-tocopherol in the LV (nmol/g)	CoQ9ox in the LV (nmol/g)	CoQ10ox in the LV (nmol/g)
c	58.8 ± 4.6	2.1 ± 0.4	248.2 ± 12.1	25.5 ± 2.3
24	50.6 ± 2.3	1.2 ± 0.2*	255.0 ± 21.5	25.0 ± 2.8
24C	49.9 ± 4.2	1.4 ± 0.2	290.1 ± 19.3	29.2 ± 1.8
24M	49.7 ± 2.8	1.6 ± 0.2	286.9 ± 29.1	26.8 ± 2.5

Values are mean ± SEM, *P < 0.05 versus c, ^#^ *P* < 0.05 versus 24.
